# Cattle Inspection and the Tuberculin-Test1Read before the semi-annual meeting of the Pennsylvania State Veterinary Medical Association, Erie, Pa., September, 1900.

**Published:** 1901-03

**Authors:** G. B. Jobson

**Affiliations:** Franklin, Pa.


### CATTLE INSPECTION AND THE TUBERCULIN-TEST.[1]

By G. B. Jobson, V.S.,
FRANKLIN, PA.

This subject of cattle-inspection, being one in which we are all more or less interested, I have thought it well to present to this meeting more for the purpose of discussion than with the hope that I may be able to offer anything that is original. In this endeavor I will confine myself to a few salient points in connection with the tuberculin-test.

What is the influence of adventitious circumstances on the initial temperature? Is the temperature of a cow or heifer in heat influenced by this fact? I can give only one instance in my experience where I have found this to be the case. A Holstein heifer, fifteen months old, whose temperature at 2 P.M. was 102.7°, went to 103° at 5 P.M., and to 104° at 9 P.M. She was tested five weeks afterward, when her highest initial temperature was 102.2° and highest post-injection temperature 101.7°. She had been brought in from pasture on the first day of the test, and consequently her normal temperature ranged higher on that day than on the preceding day. The temperature last July of a newly-served, three-year-old heifer, chased in from pasture, where she had been running with a bull, was 102.3°. The average mean temperature of the grown cows in the herd at the same hour was 102°, a rise of only three-tenths of a degree, easily accounted for by the greater difficulty experienced in driving her to the stable. Her highest post-injection temperature was 101.7°.

No doubt all of you have observed that the temperature of cattle newly stabled, at least in warm weather, runs high. The average mean initial temperature of seventeen head, young and old, taken at noon of July 16, 1900, about one hour after tying up, was 102°. These cattle were all free from tuberculosis, having been injected with tuberculin at 9 P.M. of this day. At noon next day the mean temperature of the lot was 101.6°, four-tenths of a degree lower than on the preceding day. Cattle recently dehorned, or accidentally injured, should not be tested until they have recovered from their injuries. In testing a lot of cows this summer, some of which had been dehorned two or three days previously, the result was very unsatisfactory, their temperatures both before and

[1] Read before the semi-annual meeting of the Pennsylvania State Veterinary Medical Association, Erie, Pa., September, 1900.

after injection being very irregular. A retest after their stumps were healed showed them to be all right.

Does the age of an animal influence its temperature? The mean initial temperature of ten head of cows over three years old, giving milk, taken in the month of February at 10 A.M., was 101.3°; at 3 P.M., 101.2°, and at 9 P.M., 100.9°. That of ten head of cattle under one year old, taken the same day, was, at 10 A.M., 101 2°; at 3 P.M., 101.7°, and at 9 P.M., 101.4°. On the whole lot an average of about four-tenths of a degree higher in the case of the young animals. In October, 1899, of seven cows and two bulls over two years old—in all nine head—the mean initial temperature was, at 4 P.M., 101.8°, and at 9 P.M., 101.3°. That of nine head of young stock, twelve months old and under, on the same day at 4 P.M. was 102.8°, and at 9 P.M. 102.1°, the younger cattle running nine-tenths of a degree higher than the older ones. All the above cattle were free from tuberculosis. I mention this fact because you will probably have observed far-advanced cases of tuberculosis, with the system saturated with the tubercular toxins, have generally an abnormally high temperature, and sometimes fail to show any rise of temperature after the injection of tuberculin.

Some inspectors do not recommend testing cows which are heavy with calf unless the initial temperature of the animal is abnormally high. I invariably test them rather than have an untested animal in the herd. If there should be some reaction, giving rise to a suspicion that the animal is tuberculous, she can be isolated until a retest is made. I have frequently tested cows almost due to calve and in some cases animals which have dropped their calves within a day or so after the test, and it is comparatively seldom that I have observed any with a temperature which ran very much higher than the balance of the herd.

What is the result of a retest in suspicious cases—such as give a reaction of not quite 2°? If there is no reaction on a retest, is it conclusive that the animal has not tuberculosis? I think not. Repeated injections of tuberculin closely following the primary injection, even when given in from a half more to a double dose, frequently fail to give a reaction, and apparently render the animal immune.

Five cows tested October 11, 1899, and retested January 10, 1900, failed on a retest, with double the usual dose, to give as decided a reaction as at the first test.

No. 1 on test gave a reaction of 1.8°; on retest a reaction of 1°.

No. 2 on test gave a reaction of 2.1°; on retest a reaction of none.

No. 3 on test gave a reaction of 2°; on retest a reaction of 0.7°.

No. 4 on test gave a reaction of 3.1°; on retest a reaction of 0.7°.

No. 5 on test gave a reaction of 2.3°; on retest a reaction of 1°. Several cows tested this spring and summer at intervals of three and four weeks apart, some of which at the primary test showed sharp reactions, gave comparatively low reactions following the second and third injections, not enough to condemn them as tuberculous, even with one-half more than the usual dose of tuberculin. One of these which at the primary test gave a reaction of 3.4° three weeks afterward on a test reached 0.8°; one month afterward on a second test reached 1°, and four months afterward on again being tested failed to react. Six weeks after this last retest she died after a short illness. On autopsy the right lung was found to be in the stage of red hepatization, and the left lung less engorged. Evidently pneumonia was the immediate cause of death. The tubercular lesions, which were not even extensive, were as follows: One post-pharyngeal gland enlarged to the size of a hen's egg; centre necrosed and filled with dry caseous material; caseous deposits in three of the mediastinal glands and left and right bronchial glands; circular caseous abscesses, each two inches in diameter, in the second cephalic and ventral lobes of the right lung. No other lesions were discoverable in any of the other organs.

A more satisfactory result was obtained in the case of five cows tested October 25, 1899, and retested June 4, 1900. The usual dose of tuberculin was injected at both tests. These cows, three of which were registered, were condemned on the first test, but quarantined, and milk boiled and fed to calves until owner could replace them with others. A retest was made by way of experiment. On autopsy all were found to be tuberculous, but not extensively. The record is as follows:

No. 1 test gave a reaction of 5.6°; on retest a reaction of 5.4°.

No. 2 test gave a reaction of 4.6°; on retest a reaction of 4.3°.

No. 3 test gave a reaction of 3.6°; on retest a reaction of 2.8°.

No. 4 test gave a reaction of 3°; on retest a reaction of 4°.

No. 5 test gave a reaction of 3.5°; on retest a reaction of 3.1°.

Contrasting this record with that of the five cows tested October 11, 1899, and retested January 10, 1900, if any of these cows are tuberculous, it would appear that the intervention of a longer period between the test and retest is more effectual than giving increased doses.

Sometimes it is a difficult matter to draw the line between suspicious cases which ought to have a retest and those which ought to be condemned. The reaction comes slowly, and is weak, running to 104° or thereabouts; sometimes under this mark, yet giving a reaction of over 2°. Some I have killed at the request of their owners, who wanted their herd to be above suspicion. The tubercular lesions, as a rule, are slight, and it has been my experience usually to find them in the left bronchial lymph-gland. Where a herd is tested at intervals of six months or one year tubercular lesions in reacting animals are frequently difficult to discover after the first or second test.

What is the most satisfactory method of arriving at the value of condemned animals? We may assume that in the majority of cases there will be no question as to the expediency of destroying common stock condemned as being tuberculous in preference to keeping them isolated and quarantined from the sound herd, yet when it comes to the matter of valuation there is likely to be a difference of opinion between the owner and the agent of the State Live-stock Sanitary Board. The former in many cases expects to be remunerated to the full amount allowed by the board, irrespective of the conditions of the animal. A method which I have found to work well is to get the owner clearly to understand that he can only be compensated for the present actual value, and that the value of any animal must in no case exceed twenty-five dollars for common cattle or grades, and fifty dollars for registered stock; then pick out his best animals at once, agreeing to give the limit of price for them if worth it, and if the owner is reasonable you will usually have little difficulty in agreeing on a satisfactory scale of prices for those of inferior quality. Yet I confess that the question of making a correct ante-mortem valuation of a tuberculous animal is replete with difficulty, as a post-mortem examination might reveal lesions which rendered the carcass utterly unfitted for food.

What is an animal worth the flesh of which is unfit for consumption? At the Chicago stockyards the tankage value is one cent per pound computed on the basis of the weight of the dressed carcass; in other words, a dressed carcass weighing six hundred pounds would be worth six dollars, and the offal thrown in. The average price of country hides the year round is about seven cents per pound, and weighing fifty pounds would be three dollars and fifty cents, making the total value of the animal if used in this manner nine dollars and fifty cents. The tankage value of emaciated animals is three-quarters of a cent, reducing the value accordingly.

Although there are difficulties attending the use of tuberculin as a diagnostic agent, I believe it is the only sure method by which tuberculosis can be detected and eradicated from a herd. It is true a mistake may occasionally be made and an animal destroyed in which the disease cannot be macroscopically demonstrated; but this does not often occur, and after all a microscopic examination might reval the presence of the bacilli and prove the diagnosis correct. I will cite an instance of the rapidity by which tuberculosis can be controlled by its use. In a herd of fifty head tested in October, 1899, twenty-two reacted; on testing the herd, now numbering twenty-nine, in June, 1900, only one animal reacted. An autopsy showed the animal to be only slightly affected with tuberculosis.

